# Photoreceptor PhyB Involved in *Arabidopsis* Temperature Perception and Heat-Tolerance Formation

**DOI:** 10.3390/ijms18061194

**Published:** 2017-06-05

**Authors:** Junyi Song, Qijun Liu, Biru Hu, Wenjian Wu

**Affiliations:** 1 College of Science, National University of Defense Technology, Changsha 410073, China; songjunyi@nudt.edu.cn (J.S.); ivanliuqj@nudt.edu.cn (Q.L.); wjwu67@nudt.edu.cn (W.W.); 2 State Key Lab of Nuclear, Biological and Chemical Protection for Civilian, Beijing 102205, China

**Keywords:** *Arabidopsis thaliana*, heat stress response, transcriptomic and morphological analysis, photoreceptor-phyB, interplay between abiotic stresses

## Abstract

The influence of temperature on plants is essential. However, our knowledge on the intricate regulation process underlying heat stress (HS) response in plants is limited. Recently, information about thermal sensors in vivo has begun to emerge. In this study, another primary environmental stimulus, light, was verified once again to work with temperature synergistically on plants, through the modulation of numerous biological processes. With the application of transcriptomic analysis, a substantial number of heat-responsive genes were detected involved in both light- and phytohormone-mediated pathways in *Arabidopsis.* During this process, phytoreceptor phyB acts as a molecular switch to turn on or turn off several other genes HS response, under different light conditions. Furthermore, a morphological study showed the afunction of phyB enhanced plants thermal tolerance, confirming the important role of this phytochrome in temperature perception and response in plants. This study adds data to the picture of light and temperature signaling cross-talk in plants, which is important for the exploration of complicated HS responses or light-mediated mechanisms. Furthermore, based on its influence on *Arabidopsis* thermal response in both morphological and physiological levels, phyB is a photoreceptor, as revealed before, as well as an essential thermal sensor in plants.

## 1. Introduction

With relatively weak mobility, plants have to adjust their physiological and metabolic processes according to their surroundings [1]. Light and temperature are two major environmental factors influencing plant growth and development substantially [2,3,4].

As an energy and stimulus source, light codes information into its quantity (fluence), quality (wavelength), direction, and duration, providing plants with spatial, temporal, and seasonal clues. Thus, plants can regulate multiple developmental processes throughout their lifecycle—about when to sprout, flower, and settle seeds [5]. It is relatively clear about how plants perceive light changes and transmit these signals [6]. For example, phytochromes and cryptochromes act as regulators of germination [7], flowering and hypocotyl elongation [8] according to red/far red and blue light conditions.

On the other hand, temperature is a key parameter influencing the metabolism and cell functions of living beings [9,10,11]. The impacts of temperature change are determined by intensity, duration, and rate [12]. Ambient temperature provides plants with clues about when to dormant, germinate, and swift from the vegetative growth stage to the reproductive stage [13]. However, because the responses to temperature are systematic and extremely complicated [14], our knowledge about temperature sensors in plants is quite limited [15].

For quite a long period, these two factors have been studied separately. However, physiological processes in plants are influenced by synergistic effects of multiple surrounding signals [16,17,18]. As two ubiquitous environmental factors, light and temperature work simultaneously [19] to regulate plant germination [20,21], flowering, and reproduction [22,23]. Studies have already indicated a tight relationship between light and temperature signaling pathways in plants [24,25].

Recently, more direct evidence has been found. By chasing the formation of the phyB nuclear body, Martina Legris et al. verified that phytochrome responds to both light and temperature stimulations [26]. Jae-Hoon Jung et al. reported that plant heat stress (HS) response can be altered in both transcription and morphology levels by knocking out the function of phytochrome [27].

By adopting different approaches, consistent discoveries are presented in this paper. Firstly, the expression of a substantial number of genes was detected to be activated or depressed after being treated with high temperature stress. This global monitoring identifies most HS responsive molecular elements in plants, providing us with a cornucopia of thermal sensor investigation.

Bioinformatics analysis was performed to decode the data. It revealed that various essential factors involved in plant light-signaling pathways and phytohormone-metabolic processes responded to the HS treatment. Therefore, based on the transcriptomic data acquired in this study and the information collected by bioinformatics approaches, an exciting blueprint shows that light and temperature perception pathways in plants are integrated. Meanwhile, these two signaling cascades converge and deliver messages to each other by the coordinated regulation of phytohormone metabolism.

Moreover, the bioinformatics analysis of microarray data inspired us to pay attention to the performance of photoreceptor phyA and phyB in plant HS response. It was assumed that, if these two phys participated in this process, the HS response in plants could be modified by photostimulation. In consideration of this, we pioneered the detection of transcriptional response to HS under different light conditions. The outcome of this assay revealed that light was an essential incentive, which adjusted the HS response in plants by regulating the active states of phys.

Furthermore, morphological observations on phytochrome null plants were conducted. Although Jae-Hoon Jung has already accomplished similar experiments, we are the first to report the influences of HS in *Arabidopsis* lateral root development. The observation on *phyB* mutants shows that the afunction of this photoreceptor diminished the HS effects on lateral root development and increased the survival rate of plants after HS treatment.

## 2. Results and Discussion

### 2.1. Microarray Analysis of col-0 under Heat Stress

Genome scale transcriptional analysis is essential for abiotic stress response studies in plants [12,15,28,29]. The microarray successfully detected the significant HS response in plants, with 1852 differentially expressed genes (see Table S1) between control (23 °C) and HS-treated groups (37 °C). To determine their functions, these genes were mapped according to the GO database by GOEAST. Furthermore, DAVID helped us locate these genes to established KEGG pathways (see Supplementary KEGGs and GOEASTs). These gene-ontology and functional-enrichment analysis showed that the plant responses to heat stress were involved in numerous biological and metabolic processes. The most significant response was the upregulation of *HSPs* (heat shock proteins). The accumulation of HSPs is an essential reaction to heat stress [28,30].

Some low-molecular-weight HSPs, sized about 17 to 30 kDa, are categorized as smHSPs. They prevent irreversible protein inactivation and aggregation, contributing to the development of thermal tolerance. The smHSPs are not usually expressed by vegetative tissues in the absence of heat stress. However, the protein content detection and cDNA library screening showed that particular sm*HSPs* could accumulate to over 1.0% of the total leaf or root cell protein. Therefore, the overexpression of sm*HSPs* detected in this study (see Table S1) confirmed the ongoing heat shock response.

**Figure 1:**
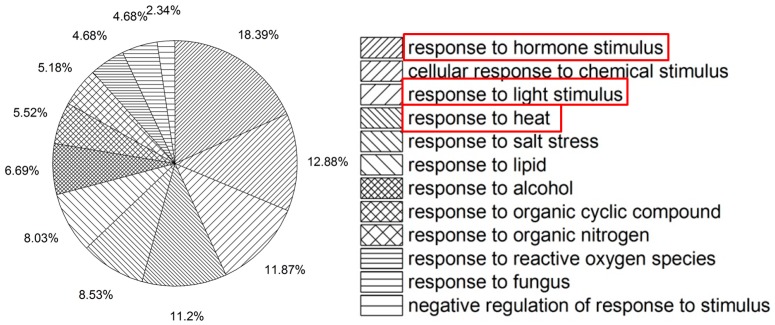
GO enrichment analysis of differentially expressed genes to HS treatment. GOEAST analysis reveals that the heat stress (HS)-induced differentially expressed genes are enriched into several biological processes (see original map in Supplemental KEGGS and GOEASTs). A statistics summary of biological processes on Level 4 (the 4th rank of GOEAST-biological_process, see Supplementary GOEAST GENE LIST) is presented here. On this level, 18.39% and 11.87% of the HS-induced genes are involved in hormone stimulus and light stimulus response, respectively. (Genes involving more than one category are double-counted. Red frames highlight the three “responses”.)

Nevertheless, what truly interests us is the HS response of light- and phytohormone-related genes. According to the functional annotation clustering data provided by DAVID and GOEAST, we found that genes that responded to heat stress were also highly enriched in a light radiation stimulus response and phytohormone signaling pathways (see Figure 1).

By driving photosynthesis and carbon assimilation, light is an essential environmental factor in plant metabolism, providing a chemical energy source for the plant’s life cycle [31]. Therefore, plant growth and development are dramatically regulated by environmental light [32]. Genes listed in Table S2 show that HS has a powerful impact on the expression of these “light-responsive” genes, including 14 transcription factors (see Table 1).

Taking DREB2 and NACs, for example, DREB2 protein, primarily activated by dehydration, high salts [33], and light [34], is reported to bind to a series responsive element. While in this study, the stress treatment is HS. Since strong radiation, high temperature, and drought are three essential ambient stimuli that happen simultaneously [35], it can be understood why the mRNA abundance of *DREB2A* was also increased here. The situation of NACs, another group of TFs, is similar to DREB2A. They answered the HS treatment, with light [36] and dehydration [37] as responsive elements. As for the *HY5* in this table, it is a more representative element playing an important role in light signaling pathways [4,16]. These demonstrate the sharing of key molecular components and the continuous information communication between light and temperature signaling cascades.

Gene ontology enrichment analysis also indicated that genes related to phytohormone-mediated pathways substantially reacted to heat stress (see Table S3), including 25 transcription factors (see Table 2). It is interesting that the expressions of most TFs were suppressed. To confirm it, qPCR was conducted on several auxin-biosynthesis and transportation genes (taking *ACTIN2* as an internal reference, the relative expression is obtained by comparing it with a control group (23 °C)).

**Figure 2:**
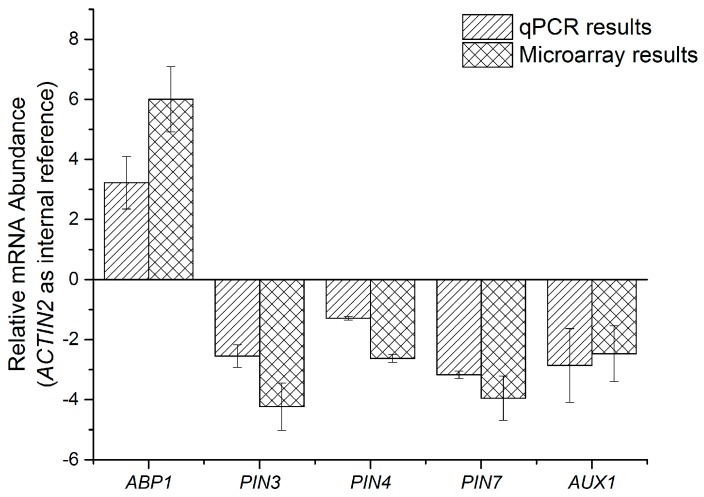
Relative expression of auxin-metabolism related genes. For both qPCR and microarray analysis, plants grown at 23 °C were collected as control group, and plants grown at 37 °C were collected as HS group. *ACTIN2* was used as an internal reference for both assays.

Data in Figure 2 verify the reliability of these two independent approaches. These genes were selected because auxin orchestrates many physiological mechanisms under heat stress [38]. Consistent with previous research, the expressions of *PIN3*, *PIN4*, *PIN7* [39], and *AUX1* [40] were repressed, as the production of auxin production is reduced during heat stress.

For the ABP1, a protein binds the phytohormone auxin with high specificity and affinity, and its role in auxin-induced processes is still under discussion [41]. However, as a good candidate for the auxin-mediated inhibition of PIN internalization [39], the enhanced *ABP1* expression and the decrease in *PINs* should have similar influence in auxin metabolism. This description of auxin-mediated genes is just a tiny fraction of phytohormones’ significant roles in stress responses [42]. It is complicated for the interaction among different phytohormones [40], as well as the feedback mechanism between hormones and their molecular regulators [43]. In addition, the synergistic effects of ambient stimuli makes the situation more intricate [44].

Therefore, it is quite difficult to explore and confirm the function of phytochromes in a single study. However, the extensive HS response of related genes of phytohormone in this study has already effectively demonstrated their essential roles during this biological process. Moreover, considering their effective reactions to ambient changes, these phytochromes should be the common molecular components of light and temperature signaling pathways in plants, acting as bridges to deliver messages between each other.

To sum up, the treatment (37 °C for 30 min) applied in this study had successfully induced the transcriptomic HS response in *Arabidopsis*. More intriguingly, it is found that differentially expressed genes induced by high temperature are enriched in two biological processes—phytohormone metabolism and light signaling. Light- and temperature-signaling processes seem to deliver information to each other through their common molecular components, phytohormones.

Furthermore, according to the enrichment analysis, several genes induced by HS were found to be involved in map04712, in the KEGG database (see Supplementary KEGGS and GOEASTs). This shows that the HS responsive elements in this study are enriched in red and far red light perception and transduction parts. To further understand how light and temperature perception in plants intervenes with each other, a qPCR analysis was conducted.

### 2.2. qPCR Analysis of Transcriptomic Response to HS under Light Conditions

To explore how light influences the transcriptomic response to HS and the function of phyA and phyB in temperature perception, we treated plants with a series of temperature and light stimuli. Although the synergistic effects of environmental stimuli on plants have been studied previously, this is the first time that white light was divided into several wave bands before being combined with HS treatment. Then, the mRNA level (*ACTIN2* adopted as internal reference) of several genes was detected by qPCR. The CCA11 [2,3] and HY5 [4,14] were chosen because they are important downstream factors of phyA and phyB in signaling cascades of circadian rhythms. Meanwhile, PIF4 and PIF5 were selected because they are basic helix-loop-helix (bHLH) phytochrome interaction factors, specifically interacting to the far-red light-absorbing Pfr form of phyB through conserved domains [45]. In addition, since we are curious about how phytohormone ethylene might be involved in the interaction of light and temperature stimuli on plants, the transcript level of *ERF7* was also examined.

**Figure 3:**
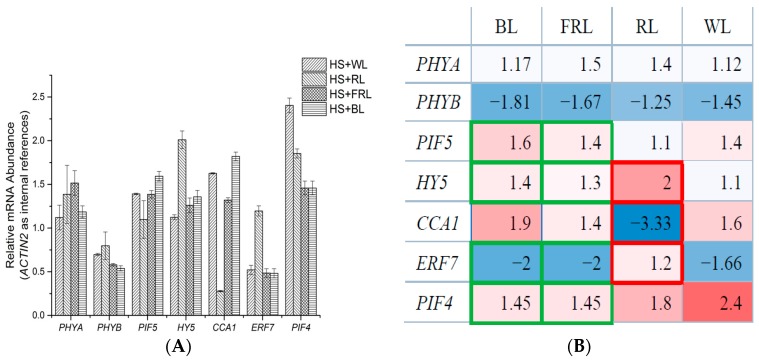
Transcriptomic response to HS, under different light conditions treatment (compared to control plants, *ACTIN2* used as internal references). Plants grown at 23 °C are chosen as control. mRNA extraction and qPCR experiments are conducted as described in the method. HS + WL: Plants in this group are treated at 37 °C for 30 min, under white light. HS + RL: Treated at 37 °C for 30 min, under red light. HS + FRL: Treated at 37 °C for 30 min, under far-red light. HS + BL: Treated at 37 °C for 30 min, under blue light. WL, white light; RL, red light; FRL, far-red light; BL, blue light. The histogram (**A**) shows the expressions of genes in different light conditions; the table (**B**) demonstrated the similarity between BL and FRL groups (green frames), and the particularity of RL group (red frames). Blue shadow means the gene expression was repressed by HS. Red shadow means enhanced.

To ensure that the microarray results can be compared, the same temperature conditions (23 and 37 °C) were settled. To test whether light conditions affect HS response in plants, four light conditions were chosen: white light (WL), red light (RL), far-red light (FRL), and blue light (BL). WL was selected so that the qPCR and microarray results could be compared under the exact same condition. By doing this, the reliability of these two approaches was verified. RL and FRL were employed because they are effective signals in triggering the transformation of the photochrome active state [46,47]. In addition, although not too many HS-induced genes are enriched in blue light signaling, the HS + BL treatment was also conducted to plants, as a valuable comparison. Under a white light condition (see Figure 3), the variation trend and amplitude of *PHYA*, *PHYB*, *PIF5*, *HY5*, *CCA1*, *ERF7*, and *PIF4* were highly consistent with microarray results, demonstrating that the outcomes of two independent experiments are credible. Making plants grow at 23 °C and WL as the control group, it was observed that several genes responded to HS diversely under different light conditions.

The results of this qPCR test can be summarized as follows: (i) The expressions of both *PHYA* and *PHYB* showed no significant difference among groups; (ii) For the other genes, light seemed to disrupt their heat response substantially. The expression of *PIF4* was obviously enhanced by heat only when it was under WL or RL. The expression of *ERF7* stayed unchanged under RL, but suppressed severely under WL, BL, and FRL. The expression of *CCA1* decreased sharply under RL. *HY5* experienced a significant expression enhancement under RL; (iii) Under BL and FRL conditions, the expressions of *PIF4*, *PIF5*, *ERF7*, and *HY5* were extremely similar and quite different from the RL situations.

Comparison among different groups shows that the active state transition of phyA and phyB led to diverse HS responses. These two phytochromes mediate plant physiological mechanism processes by switching between active and inactive forms, according to the change in ambient light conditions: Upon absorption of R, phytochromes are converted from the biologically inactive Pr form into the active Pfr form, whereas FR irradiation converts Pfr back to Pr [48]. Experimental results in this study demonstrated that these phytochromes performed more than just light receptors. Comparison between HS + RL and HS + FRL groups directly revealed that phytochromes regulated the HS response of their downstream components by self-adjusting their own biological active states. This deduction can be further certified when we take a look at situations under blue light and far red light. It is clear that the expression of genes under BL and FRL conditions were very similar (data circled with green border). In the two situations, most of phyA and phyB were in the biological inactive Pr form. The difference between these two groups is whether blue light receptor cryptochromes were activated or not. The data shows that the activation of cryptochromes alone does not alter the HS responses of *HY5*, *PIF4*, and *PIF5*, which are downstream components of these blue light receptors [49]. Therefore, though the function of cryptochromes cannot be completely excluded, the effects of a phytchrome’s active state should be dominant during plant HS perception and response.

This is not the first time that phytochromes have been reported to involve temperature perception and regulation. Karayekov et al. reported that phytochrome B mediates plant de-etiolation after the HS treatment [47]. Feng Wang et al. declared that far-red (FR) and red (R) light, perceived by phytochrome A (phyA) and phyB, positively and negatively regulate cold tolerance, respectively, in tomato (*Solanum lycopersicum*) [50]. The switch of the active state of phyB was also observed by Martina Legris et al., when they treated *Arabidopsis* with various temperatures [26]. However, this study firstly reported how these photoreceptors influence the HS responses of their downstream factors, in different light wave bands. As a light stimulus receptor, phytochromes are also crucial molecular sensor for ambient temperature perception and response. Further, the substantial effects of phyB on plant HS response were verified by the following phenotypic observation.

### 2.3. Effects of HS on Different Arabidopsis Genotypes

Recently, an increasing amount of research has shown that some light responsive-components are not exclusively activated or repressed by photostimulation [32]. Meanwhile, the transcriptomic analysis in this study also demonstrates the sharing of common components by light and temperature signaling pathways in plants.

**Figure 4:**
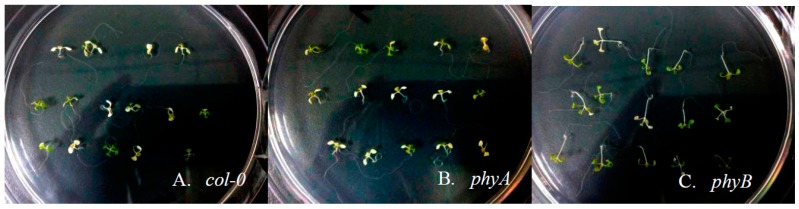
Survival analysis of different *Arabidopsis* genotypes under HS treatment. After treatment at 37 °C for three days, about 44% and 40% *col-0* and *phyA* seedlings still survive (see (**A**,**B**)); However, the survival rate of *phyB* is more than 70% (see (**C**)).

To further verify this, HS treatment (37 °C for three days) was treated on 6-day-old seedlings of *col-0*, *phyA*, and *phyB*. After this relatively continuous HS treatment, these genotypes exhibited different performances in morphological level: only about 44% and 40% *col-0* and *phyA* withstood HS treatment, while more than 70% *phyB* stayed alive after this harshness (see Figure 4). In other words, the absence of phyB function seems enhanced the thermal tolerance substantially in plants.

**Figure 5:**
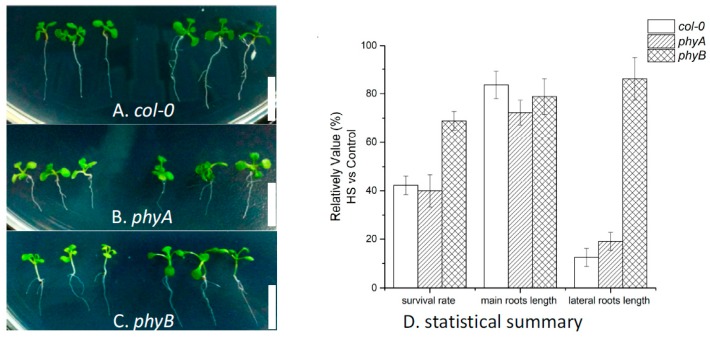
Root development of three genotypes of *Arabidopsis* (control individuals were placed on the right, treated ones left), and statistical summary for phenotype analysis. After treatment at 37 °C for three days, the main roots lengths of all three genotypes were suppressed to a similar degree. Meanwhile, the lateral root development of *col-0* and *phyA* seedlings are severely inhibited (see (**A**,**B**,**D**)). However, the lateral roots of *phyB* showed superior development (see (**C**,**D**)). Scale bars, 1 mm.

Based on the observation of survival individuals, it is found that root development of plants was impacted to different degrees. For *col-0*, the lengths of their main roots were 10–15% shorter after 3-day HS treatment. For *phyA* and *phyB* mutants, their main roots lengths were suppressed by 20–25%. Therefore, the HS influences on the main roots of these three *Arabidopsis* genotypes were similar. However, the HS responses of lateral root developments were quite diverse. The wild-type *col-0* hardly developed lateral root when grown under HS, in contrast to the well-developed roots under control condition (see Figure 5A). For *phyA*, though the afunction of phyA already impedes its lateral development in a normal temperature condition, the HS treatment made the situation even worse: The number and length of lateral roots decreased significantly (see Figure 5B). Conversely, the lateral roots of *phyB* grew quite well even treated with extremely high temperature (see Figure 5C).

These results certificated the essential function of phyB in plant HS response. This kind of phytochrome enhanced the thermal tolerance of *Arabidopsis* individuals by boosting their lateral root development, increasing their survival rate under long-term high temperature stress. This regulatory effect of phyB during plants HS response could be achieved by controlling its downstream components, including PIF4, PIF5, and HY5. Take PIF4 and PIF5, for instance. Mutant experiments have proved them to be crucial for *Arabidopsis* hypocotyl elongation, during high temperature stress [51,52]. However, as basic helix-loop-helix (bHLH) transcription factors that tightly interact with the light-activated Pfr form of phyB [53], their performance during temperature response must be substantially altered by ambient light stimulus. As another TF tightly related with plant light signaling, HY5 mutants have been reported to participate in not only the regulation of *Arabidopsis* hypocotyl elongation [54,55] but also the formation of stress tolerance. Nawkar et al. reported that the mutation of HY5, which negatively modulates the unfolded protein response, leads to a higher tolerance to endoplasmic reticulum (ER) stress [56]. In their study, HY5 acts as a repressor of stress-tolerance formation. This provides a clue to explaining our observation. Because of the absence of phyB function in *phyB* mutants, the effect of its downstream component HY5 was also abolished or disturbed to some extent during HS treatment. Accordingly, these mutants acquired a higher tolerance to ER stress caused by HS in this study, with a higher survival rate and better-developed lateral roots. This is also consistent with the qPCR results that the expression of HY5 increased more significantly in RL than in BL or FRL, indicating that biological active phyB is essential for HS-induced HY5 enhancement. In short, the deletion of phyB function by either mutation or photostimulation will dismiss the repressor role of HY5 on plant stress-tolerance formation.. Therefore, the HS response and thermal tolerance in plants can be substantially regulated by phyB, through its adjustment of downstream molecular components.

## 3. Materials and Methods

### 3.1. Plant Materials and Growth Conditions

*Arabidopsis* (*Arabidopsis thaliana*) genotype *col-0* was used for all experiments. Sterilized seeds grew in Murashige & Skoog solid medium within glass plates. After 72 h of vernalization at 4 °C in dark condition, plates were transferred to the growth camber (an ambient temperature of 23 °C, a relative humidity of approximately 70%, and a photoperiod of 16 h of light, approximately 15.67 W/m^2^, alternating with 8 h of darkness).

### 3.2. Heat Stress Treatment

Ten-day old plants were performed with heat stress treatment through a similar method as reported elsewhere. After 10 days, seedlings plants were separated into two groups—the HS-treated group (at 37 °C for 30 min) and the control group (without HS), with three biological replications for both groups. After stress treatments, whole plant samples were collected, promptly frozen in liquid nitrogen for a subsequent microarray, and qPCR experiments.

For morphological analysis, 6-day-old seedlings were treated with a long period of HS treatment (at 37 °C for 30 days).

### 3.3. Light Conditions Adopted during HS Treatment

For the different HS-treated groups, the high temperature is the same (at 37 °C for 30 min). The light conditions are represented as follows: WL—15.62 W/m^2^, RL—19.50 W/m^2^, FR—19.81 W/m^2^, and BL—4.13 W/m^2^.

### 3.4. Microarray Hybridization and Analysis

We employed the Affymetrix GeneChip (Affymetrix, Santa Clara, CA, USA) *Arabidopsis* ATH1 genome array designed specifically for monitoring gene expression in *Arabidopsis*. Total RNA extraction and data analysis methods have been described in previous studies [15].

### 3.5. qPCR Analysis

For qPCR analysis, the plants from two groups—the HS-treated group and the control group (without HS)—were sampled for RNA extraction. The RNA extraction method used in both microarrays and qPCR analysis were consistent. After being treated with Dnase I, RNA samples were used for cDNA synthesis (Thermo, Vilnius, Lithuania). Then, qPCR was performed using the LightCycler^®^ 480 SYBR Green I Master (Roche, Mannheim, Germany) with 10 pmol of each primer in 10 mL, and the reactions were run on a LightCycler^®^ 480 (Roche). For this verification, biological triplicate *col-0* samples were used. Relative expression was determined by the using of *ACTIN2* (AT3G18780). As reported to be highly expressed with minimal variation across the different treatments in the microarray data [23], *ACTIN2* is widely used as one stable internal reference gene in the studies of heat-induced transcriptomic response in plants. The results of qPCR were calculated and displayed using LightCycler^®^ 480 SW 1.5 software. For qPCR analysis, gene-specific primers were designed using the Primer 4 program (see primers in Table S4).

## 4. Conclusions

Light and temperature are two crucial environmental cues and stresses for plant growth and regulation. In the natural world, these two factors always act on plants simultaneously. It is the fact that germination, flowering or seed-settling happens only if a suitable ambient temperature and light intensity work together (other elements such as air humidity are also essential). Scientists have now begun to explore how light and temperature manipulates plants’ physiological and morphological parameters synergistically rather than separately.

In this study, three independent experiments were carried out. The results indicate that the perception and signaling pathways of light and temperature in plants are connected.

The expression of several molecular elements, which were involved in light response, was successfully induced by HS treatment (see Table 1, microarray data). By GOEAST and KEGG analysis, they were found to be enriched in red and far red light regulated processes. This is the initial evidence that lead us to consider the potential interaction between light and temperature signaling pathways. Further, the expression of a series genes related to phytohormone metabolism answered HS treatment. Since the phytohormone regulation is so important for plants to encounter with abiotic stresses, their extensive reaction to HS is not strange. The inspiring thing is that many of these HS-induced phytochrome genes have been confirmed to respond to light stimulus. Therefore, although the relationship between light and temperature signaling pathway in plants has been discussed recently, the transcriptomic analysis here provides us with a more direct clue that they tightly and extensively interact with each other. Moreover, phytohormones and related elements could be potential common components of these two signaling cascades.

To further verify the deduction above, we pioneered the idea to treat *Arabidopsis* with HS under different light conditions. The transcriptional response examined by qPCR demonstrated that the transform of phytochromes active state has an essential influence on the HS response of their downstream factors. Besides, consistent with microarray data, the comparison among RL, FRL, and BL groups indicates that R/FR signaling pathway is more active during the HS reaction. Hence, two phytochromes, phyA and phyB, are very likely to take part in a plant’s temperature perception and response through the regulation of related phytohormone factors.

At last, a morphological observation was conducted to verify the outcomes of transcriptional level. After a three-day HS treatment, phyB mutants exhibited much stronger heat-tolerance, with higher survival rates. What is more, it was also firstly reported that the HS damage of lateral development can be relieved in phyB mutants. This clearly points out the essential function of phyB during HS response and what happens when its role is absent. As discussed in phenotypic analysis, the significant role of this photoreceptor is likely to be formed by the regulation of its downstream molecular components, such as PIF4, PIF5, and HY5. By modulating the performance of these components, phyB carries out its power in HS signal perception and transduction.

Based on these three independent experiments, the existence of light and temperature signaling correlation in plants is firmly verified. It is such an exciting idea because a better understanding of the complicated HS response mechanism in plants may be obtained. Although the information can flow in other pathways, we are at least more capable of understanding how an ambient temperature signal is transduced through the relatively known light signaling cascades. Further, as a powerful photoreceptor, phyB seems to be a sensitive sensor to HS signals as well, similar to *Arabidopsis* and *Solanum lycopersicum* reported by Karayekov [47] and Wang [50], respectively. Exploring of phyB HS response might work toward finding this mysterious plant thermal sensor.

## Figures and Tables

**Table 1:** Transcription factors of light signaling pathways involved in heat stress response (positive “Fold Change” means upregulated after HS; negative means downregulated). These TFs (transcription factors) indicate that light signaling pathways also respond to HS to some extent.

No.	Gene Title	Fold Change
1	*TEM1* (*TEMPRANILLO 1*); transcription factor	−4.88839
2	*RGA1* (*REPRESSOR OF GA1-3 1*); protein binding/transcription factor	−2.82099
3	*GAI* (*GIBBERELLIC ACID INSENSITIVE*); transcription factor	−2.27883
4	*PAT1* (*phytochrome a signal transduction 1*); signal transducer/transcription factor	−2.02137
5	*HY5* (*ELONGATED HYPOCOTYL 5*); double-stranded DNA binding/transcription factor	2.29793
6	*NAC2*; transcription factor	3.5822
7	*MYB4*; DNA binding/transcription factor	3.60975
8	*ANAC13* (*Arabidopsis thaliana NAC domain protein 13*); transcription factor	3.86913
9	*GA3OX1* (*GIBBERELLIN 3-OXIDASE 1*); gibberellin 3-β-dioxygenase/transcription factor	4.73567
10	*ATNFXL1* (*Arabidopsis thaliana NF-X-LIKE 1*); protein binding/transcription factor/zin	5.01115
11	*DREB2A*; DNA binding/transcription activator/transcription factor	16.786
12	*AT-HSFA7A*; DNA binding/transcription factor	97.0821
13	*AT-HSFA7B*; DNA binding/transcription factor	121.056
14	*ATHSFA2*; DNA binding/transcription factor	248.051

**Table 2:** Phytohormone metabolism transcription factors involved in heat stress response (positive “Fold Change” means upregulated after HS; negative means downregulated). These TFs implicate that phytohormones play essential roles in heat stress response in plants.

No.	Gene Title	Fold Change
1	*MYC2*; DNA binding/transcription activator/transcription factor	−11.9908
2	*BT2* (*BTB AND TAZ DOMAIN PROTEIN 2*); protein binding/transcription factor	−8.87107
3	*ERF11* (*ERF DOMAIN PROTEIN 11*); DNA binding/transcription factor/transcription repressor	−8.04081
4	*MYB73* (*MYB DOMAIN PROTEIN 73*); DNA binding/transcription factor	−7.82007
5	*TEM1* (*TEMPRANILLO 1*); transcription factor	−4.88839
6	*RAV1*; DNA binding/transcription factor/transcription repressor	−4.52717
7	*ATAIB* (*ABA-INDUCIBLE BHLH-TYPE TRANSCRIPTION FACTOR*); DNA binding/transcription factor	−4.26839
8	*AS1* (*ASYMMETRIC LEAVES 1*); DNA binding/protein homodimerization/transcription factor	−3.37803
9	*MYBL2* (*Arabidopsis MYB-LIKE 2*); DNA binding/transcription factor	−3.15927
10	*MYB28* (*MYB DOMAIN PROTEIN 28*); DNA binding/transcription factor	−3.15868
11	*ERF8*; DNA binding/transcription factor/transcription repressor	−3.03177
12	*RGA1* (*REPRESSOR OF GA1-3 1*); protein binding/transcription factor	−2.82099
13	*AtTCP14* (*TEOSINTE BRANCHED1, CYCLOIDEA and PCF (TCP) 14*); transcription factor	−2.73614
14	*ERF7* (*ETHYLINE RESPONSE FACTOR 7*); DNA binding/protein binding/transcription factor	−2.47281
15	*EIL1* (*ETHYLENE-INSENSITIVE3-LIKE 1*); transcription factor/transcription regulator	−2.37976
16	*GAI* (*GIBBERELLIC ACID INSENSITIVE*); transcription factor	-2.27883
17	*AtMYB47* (*MYB DOMAIN PROTEIN 47*); DNA binding/transcription factor	-2.14196
18	*RAP2.7* (*RELATED TO AP2.7*); DNA binding/transcription factor	-2.10654
19	*IAA2* (*INDOLE-3-ACETIC ACID INDUCIBLE 2*); transcription factor	-2.09408
20	*HY5* (*ELONGATED HYPOCOTYL 5*); double-stranded DNA binding/transcription factor	2.29793
21	*CRF6* (*CYTOKININ RESPONSE FACTOR 6*); DNA binding/transcription factor	2.87763
22	*MYB4*; DNA binding/transcription factor	3.60975
23	*GA3OX1* (*GIBBERELLIN 3-OXIDASE 1*); gibberellin 3-β-dioxygenase/transcription factor	4.73567
24	*MYB43* (*MYB DOMAIN PROTEIN 43*); DNA binding/transcription factor	4.94925
25	*EPR1* (*EARLY-PHYTOCHROME-RESPONSIVE1*); DNA binding/transcription factor	7.39638

## Supplementary Materials

Supplementary materials can be found at www.mdpi.com/1422-0067/18/6/1194/s1.
