# Supplementary figures and images for: Photoreceptor PhyB Involved in Arabidopsis Temperature Perception and Heat-Tolerance Formation
Source: Int J Mol Sci. 2017 Jun 5;18(6):1194. doi: 10.3390/ijms18061194 (PMC5486017; doi:10.3390/ijms18061194)

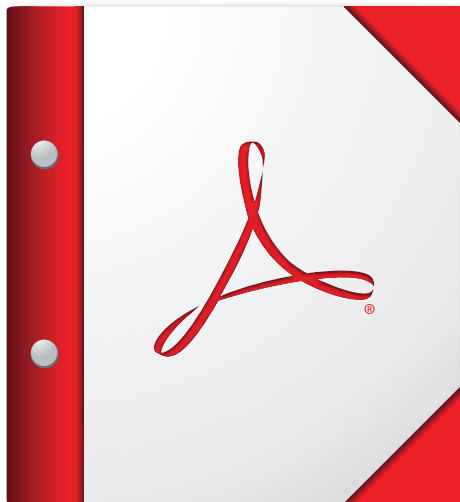

为获得最佳体验，请在 **Acrobat 9、Adobe Reader 9**  
或更高版本中打开此 **PDF** 包。

[立即购买 Adobe Reader !](#)

Supplement: Supplementary file 1 [file ijms-18-01194-s001.zip › Supplementary KEGGs and GOEASTs.pdf]
